# Organic fertilization improves soil aggregation through increases in abundance of eubacteria and products of arbuscular mycorrhizal fungi

**DOI:** 10.1038/s41598-021-91653-x

**Published:** 2021-06-15

**Authors:** Veronika Řezáčová, Alena Czakó, Martin Stehlík, Markéta Mayerová, Tomáš Šimon, Michaela Smatanová, Mikuláš Madaras

**Affiliations:** 1 Crop Research Institute, Drnovská 507, Prague 6, Czech Republic; 2 Central Institute for Testing and Supervising in Agriculture, Hroznová 63, Brno, Czech Republic

**Keywords:** Ecology, Microbiology

## Abstract

An important goal of sustainable agriculture is to maintain soil quality. Soil aggregation, which can serve as a measure of soil quality, plays an important role in maintaining soil structure, fertility, and stability. The process of soil aggregation can be affected through impacts on biotic and abiotic factors. Here, we tested whether soil management involving application of organic and mineral fertilizers could significantly improve soil aggregation and if variation among differently fertilized soils could be specifically attributed to a particular biotic and/or abiotic soil parameter. In a field experiment within Central Europe, we assessed stability of 1–2 mm soil aggregates together with other parameters of soil samples from differently fertilized soils. Application of compost and digestates increased stability of soil aggregates. Most of the variation in soil aggregation caused by different fertilizers was associated with soil organic carbon lability, occurrence of aromatic functional groups, and variations in abundance of eubacteria, total glomalins, concentrations of total S, N, C, and hot water extractable C. In summary, we have shown that application of compost and digestates improves stability of soil aggregates and that this is accompanied by increased soil fertility, decomposition resistance, and abundance of total glomalins and eubacteria. These probably play significant roles in increasing stability of soil aggregates.

## Introduction

The Earth’s soils provide many critical functions and services. Soil is the largest terrestrial carbon sink^1^, for example, and it allows the production of human food through the growth of plants upon and within it. For soil to perform these functions it needs to maintain its structural stability, namely the arrangement of soil particles into aggregates and associated pore networks^2^. Soil aggregates are important for improving soil fertility and porosity, minimizing erodibility, and endowing good agronomic productivity^3^. Aggregation is thus a crucial soil property that affects several processes important to soils’ productive capacity and environmental quality, and it is a central aspect of soil sustainability in agroecosystems^4^.

Soil aggregation can be altered by management strategies directly or indirectly through impacts on biotic and abiotic factors. Alternative agricultural models based on ecologically sound principles, including to apply organic matter such as compost and exclude the use of synthetic pesticides and fertilizers, can help sustain soil quality and its functions^5^. There are various ecological principles that can be applied, however, in order to do so successfully, it is necessary to understand their influence on soil aggregation and on the factors that affect that aggregation. For example, no-tillage and manure application increased soil aggregation and improved the quality of soils^6–8^, but the agents underlying this effect are not well known. Manure application may, e.g., increase soil aggregation through affecting bacterial and fungal community structure^8^.

Although we know that the main factors influencing soil aggregation are soil texture, soil fauna, soil microorganisms, roots, inorganic binding agents, and environmental variables^9,10^, the roles of individual factors and their interactions in the formation and stabilization of soil aggregates remains insufficiently studied. A quantitative understanding of the contribution of various soil biota groups to soil aggregation is required to predict possible consequences to soil biodiversity^11^. However, as it is soil type and climate dependent, more efforts in this respect are at present needed so that we can draw general conclusions.

Among biotic factors, mainly bacteria and fungi, and especially arbuscular mycorrhizal fungi (AMF; Glomeromycotina^12^)^11,13–15^ importantly influence the stability of soil aggregates^16–18^. AMF and bacteria are important producers of extracellular polymeric substances that bind soil particles together^19–21^, maintain ambient humidity, and serve as a reserve of carbon and mineral nutrients^3^. Glomalins are AMF products of particular importance in the formation and stabilization of soil aggregates^22,23^. In addition, AMF produce hyphae that entangle particles and hold them together^24^. Because fungal hyphae and polysaccharide products of fungi and bacteria cannot persist in the soil for long periods of time, their effects on aggregate stability are considered temporary^25^. To improve soil aggregate stability in agroecosystems and to do so sustainably probably depends also on the ability of organic fertilizers to produce humic substances^26^, decomposition-resistant mixtures containing a variety of organic compounds, many of them based on a motif of aromatic nuclei^27,28^.

When studying the influence of different types of management on soil aggregation, assessing the water stability of soil aggregates (i.e., the ability of those aggregates to resist disintegration by water) can provide reliable information on soil quality. It is very time-consuming however, to measure this parameter. If easily measurable indicators of soil aggregate stability can be selected among soil parameters, these could provide a user-friendly tool for assessing soil quality.

We asked the following questions:

(i) Is there significant variation in soil aggregate stability under the influence of different types of fertilizers?
(ii) If yes, then what explains the variation recorded among the differently fertilized soils? Could the variation be specifically attributed to particular biotic and/or abiotic soil properties?

## Results

### Effect of fertilization on stability of soil aggregates

**Figure 1:**
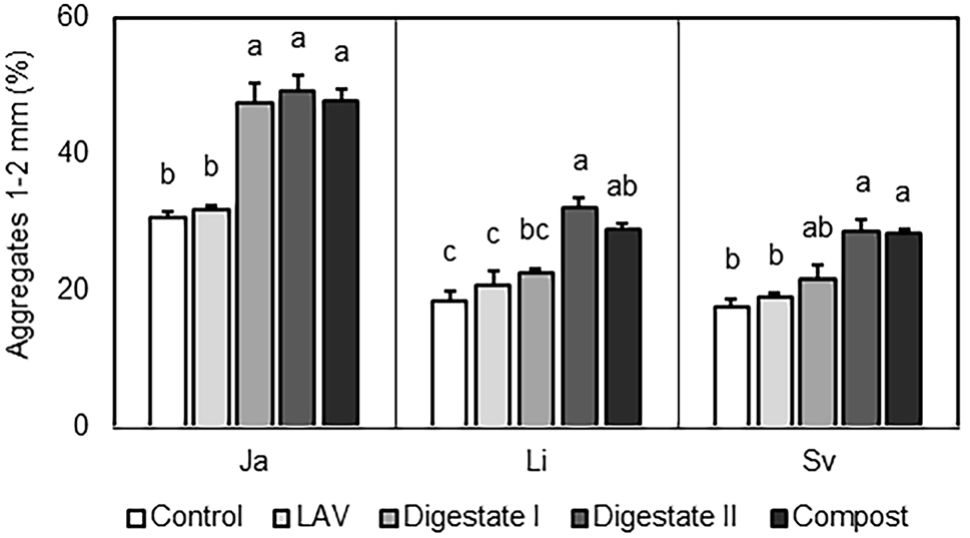
Stability of 1–2 mm soil aggregates assessed at three localities in the Czech Republic (Ja, Jaroměřice nad Rokytnou; Li, Lípa; Sv, Hradec nad Svitavou) as affected by fertilizer types (control, non-fertilized soil; LAV; digestate I; digestate II; compost). Bars represent means; whiskers show standard errors (n = 4). Different letters above individual bars indicate significant differences between means at *p* < 0.05 within the locality.

Type of fertilizer significantly affected the stability of soil aggregates (Table 1). Soil aggregates stability was consistently greater following compost and digestate II application, as well as following digestate I application at locality Jaroměřice. Digestate I application had no effect on the stability of soil aggregates at the other two localities, Lípa and Svitavy (Fig. 1). As a result, there was significant interaction between locality and type of fertilizer (Table 1). Aggregates stability was not affected by application of LAV (Fig. 1). These results are in accordance with earlier findings obtained in spring 2018, 2019, and 2020 and in summer 2018 (not evaluated within this paper).

**Table 1:** Significances of the effects of locality, type of fertilizer, and interaction of the two on stability of 1–2 mm soil aggregates as revealed by two-way ANOVA.

Dependent variable	Statistic	Locality	Type of fertilizer	Locality × type of fertilizer
Aggregates (1–2 mm)	*P*	< 2e × 10^−16^	0.08 × 10^−12^	0.04
	*F-value*	147.96	37.66	2.32

### Effect of fertilization on soil parameters

The assessed soil parameters were significantly correlated with the factor, type of fertilizer (Monte Carlo permutation test, *pseudo-F* = 3.7, *p* = 0.002), which explained about 21.4% of the soil parameters data variation (Supplementary Fig. S2 online). Especially increased abundance of eubacteria, HWC, C, N, S, and glomalins seems to be positively correlated with the application of compost (Supplementary Fig. 3 online). HWC showed positive correlation with the application of digestate II (Supplementary Fig. S3 online).

### Soil parameters as explanatory variables for soil aggregation

**Figure 2:**
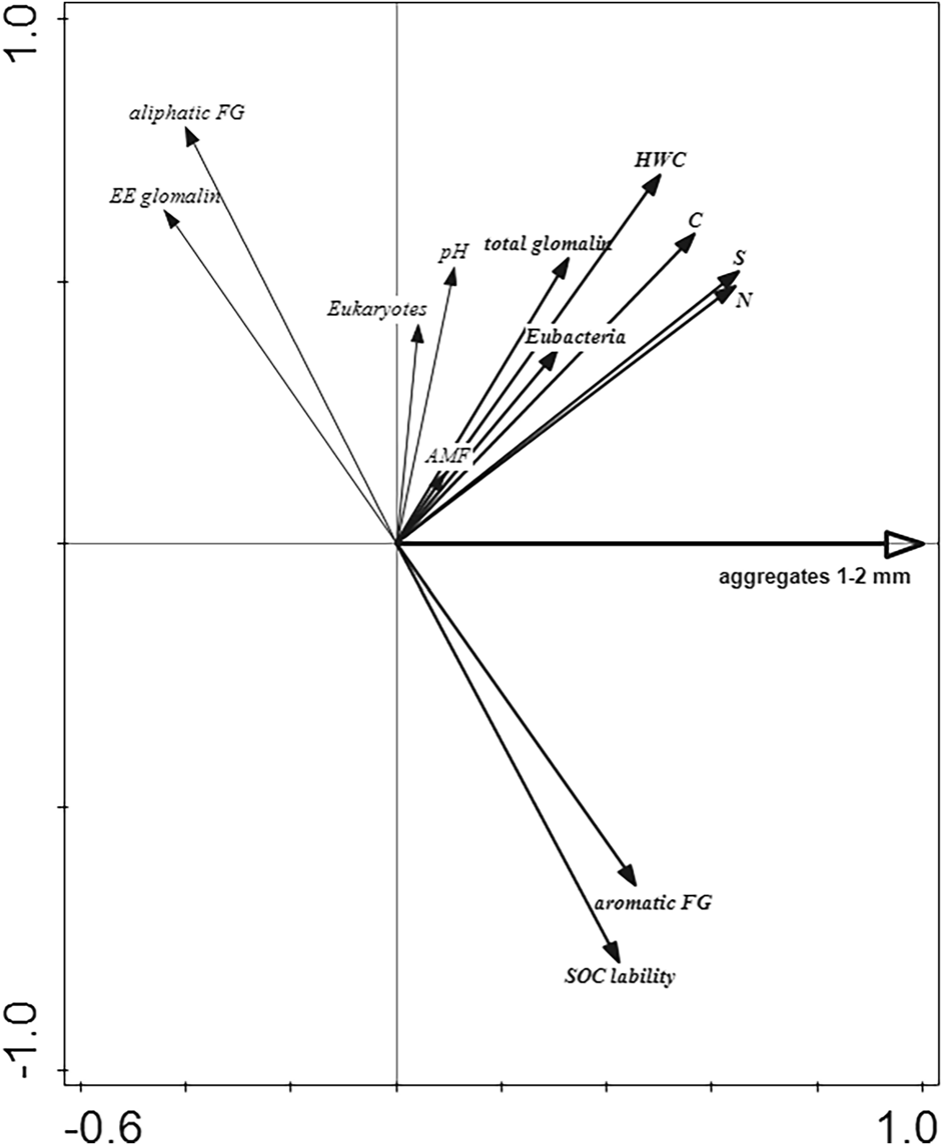
Ordination diagram showing results of redundancy analysis testing how well variation in the assessed soil parameters could be explained by the stability of 1–2 mm soil aggregates. Soil parameters positively and significantly (*p* < 0.05) associated with the predictor are shown in bold. Angle between predictor arrow and particular soil parameter arrow approximates linear correlation between the two variables (proportional to the cosine of the angle). The first (horizontal) axis explains 18.3% of the total variation in soil parameters. AMF, arbuscular mycorrhizal fungi; FG, functional groups; HWC, hot water extractable carbon; EE glomalin, easily extractable glomalins; SOC lability, soil organic carbon lability.

When scrutinizing the explanatory power of the soil parameters regarding the stability of soil aggregates, 18.3% of the variation in soil parameters data was explained by soil aggregates stability (Monte Carlo permutation test, *pseudo-F* = 13%, *p* = 0.002; Fig. 2).

As can be seen from the T-value biplot derived from this analysis, the stability of soil aggregates was positively correlated with increased abundance of eubacteria, concentration of total glomalins, S, C, N, and HWC, and occurrence of aromatic functional groups of organic matter, as well as with decreased SOC lability (Fig. 2 and Supplementary Fig. S4 online).

## Discussion

We detected significant differences in the variations of soil aggregates stability among the differently fertilized soils (Fig. 1). This means that it provides consistent biological information within the given soil context. In particular, the application of compost had a significant positive effect on the stability of soil aggregates. Bipfubusa et al.^29,30^, respectively, had reported that the addition of composted paper mill sludge and composted manure to soil had increased macroaggregate stability in a manner that is consistent with our findings. The application of digestates also had a positive effect on soil aggregation, which is consistent with the sparse data in the literature where, for example, Gielnik et al.^31^ report larger aggregates formation after applying digestate to clay-rich soil.

**Figure 3:**
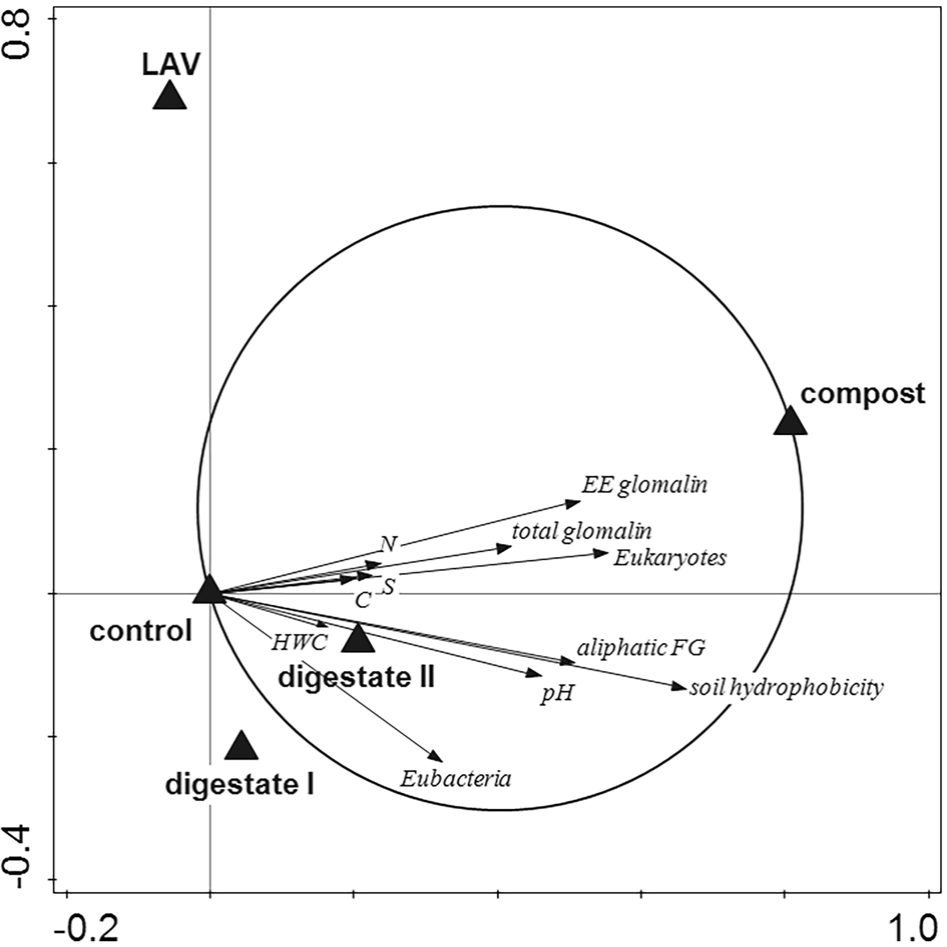
T-value biplot for the relationships between the soil parameters and the compost application. The arrows fully falling within a Van Dobben circle indicate a significant positive (*P* < 0.05) relationship between a soil parameter and the predictor. Soil parameters located outside the circles are not displayed, although they were included in the analysis. Functional groups (FG); hot water extractable carbon (HWC); easily extractable glomalins (EE glomalin); SOC (soil organic carbon) lability.

In our study, the application of compost to the soil increased, among other factors, the abundance of eubacteria and glomalins (Fig. 3), which could be the agents causing improved stability of soil aggregates^11,32^. We observed both eubacteria and glomalins to be positively associated with stability of soil aggregates (Fig. 2 and Supplementary Fig. S4 online).

Martens^26^ reports that improved soil aggregation in agroecosystems is probably dependent also on the ability of organic fertilizers to produce humic substances. This is supported by our finding of positive association of the factors decomposition-resistant SOC and the occurrence of aromatic functional groups with both the compost applications (Fig. 3) and the stability of soil aggregates (Supplementary Fig. S4 online). Bipfubusa et al.^29^ even state that in compost-amended soil, humic substances played a greater role in the stabilization of soil aggregates than did fungi.

Although it is not possible without experimental verification to demonstrate that these factors are direct agents involved in stabilizing soil aggregates, all soil factors (both biotic and abiotic) found in our study to be associated with soil aggregation (Supplementary Fig. S4 online) can be considered as suitable indicators of soil aggregation and used in creating an improved tool for soil quality assessment.

Although an influence of AMF on soil aggregation is widely mentioned in the literature^11,15,32,33^, we observed no direct relationship between AMF abundance and soil aggregation (Fig. 2). This may be due to the small variability of AMF abundance in the tested soils, as evidenced by there being no correlation between AMF and type of fertilizer (Supplementary Fig. S2). In our conditions, the effect of AMF on soil aggregation was therefore probably indirect through the production of glomalins, which is probably not related to AMF abundance. It can be a species-specific process, for example, because AMF species differ in their contributions to soil aggregation^15^. It is also not entirely clear whether or not AMF produce glomalins^15^.

In conclusion, soil management that included various organic and mineral fertilizers improved soil quality with the application of compost and digestates by improving soil aggregation. Furthermore, our data support the notion that increasing stability of soil aggregates in fertilized soils of Central Europe is related to increased abundance of soil eubacteria, glomalins, soil fertility, and resistance to decomposition. These factors explained most of the variability of the aggregate stability and/or aggregate formation in this study. This means that these factors are suitable as indicators of soil quality and can provide an alternative to monitoring tools currently in use for examining stability of soil aggregates. The assessed increase in the abundance of soil microorganisms and/or their products due to compost application, which also correlated with SAS, suggests that the combine use of microbial inoculation and compost application could be a promising approach to restoring degraded agricultural land. The unproven relationship between the total abundance of AMF and the apparent correlation of their products (glomalins) with SAS shows that total abundance of AMF determined by real-time PCR may not indicate the abundance of fungal hyphae or the amount of glomalins produced. In order to better understand the exact mechanisms through which these organisms achieve improvements in soil aggregation, it is necessary to focus on their individual taxa and their interactions next time. Research into the interactions, not only of biotic-biotic but also of biotic-abiotic factors, should be the subject of further research in this regard.

## Materials and methods

### Field experiment and sampling

Analyses of all parameters were performed on soil samples taken from plots of a crop-rotation field experiment established in 2011 at three localities of the Czech Republic’s Central Institute of Supervising and Testing: Jaroměřice nad Rokytnou, Lípa, and Hradec nad Svitavou. The localities are characterized in Table 2. Soil samples were taken at each locality from five treatments involving various types of management utilizing different types of mineral (mixture of ammonium nitrate and finely ground limestone—LAV, Lovochemie a.s., 27% N) and organic amendments (digestate I, digestate II—barnyard manure, hay, and compost; see experimental design in Supplementary Fig. S1 online). Unfertilized control was established. Fertilizer application rates were derived according to the needs of the cultivated crop for N and set so that the basic dose of N fertilization was 150 kg N ha^−1^ for LAV and digestates, from which N is released rapidly, and 300 kg N ha^−1^ for compost, from which N is released slowly. For all localities, fertilizers originated from the same sources. Digestates were produced during anaerobic biomass fermentation in biogas plant as by-product. The substrates used for the production of biogas consisted of corn silage and cattle slurry (digestate I) and corn silage, pig slurry, barnyard manure and hay (digestate II). Registered compost is produced by homogenization and composting of biodegradable substances, from urban greenery, waste from distilleries and forestry a sewage sludge (for further details see Supplementary Table S1 online). Field trial management included conventional tillage and a 6-year crop rotation in sequence potatoes/winter wheat/silage corn/spring barley/oilseed rape/winter wheat.

**Table 2:** Characteristics of the studied localities.

	Jaroměřice nad Rokytnou	Lípa	Hradec nad Svitavou
GPS coordinates	49.0997961N, 15.8762206E	49.5632089N, 15.5381272E	49.7312900N, 16.5038400E
Altitude (m)	425	505	460
Annual mean temperature (°C)	8.0	7.5	6.5
Annual mean precipitation (mm)	481	594	624
Soil type	Brown earths	Cambisols	Brown earths
Soil class	Clay loam	Sandy loam	Sandy loam

The design was based on 10.82 × 3.74 m experimental plots in 12 replicates (four replicates per locality; Supplementary  Fig. S1 and Table S2 online). Soil samples were collected from the upper soil layer (0–7 cm) in summer 2019 using field shovel. Altogether, 60 samples (12 samples of approximately 2 kg soil for each of 12 replicates per each treatment) were collected.

### Sample processing

Soil samples were air-dried, homogenized, then divided into two portions. The first portion, fine soil (< 2 mm) obtained by sieving through a 2-mm sieve, was used for assessing abundances of three groups of soil biota (AMF, eukaryotes including protists, and eubacteria); pH; hot water extractable C (HWC); concentrations of N, C, and S; and characteristics of soil organic matter. A fraction with soil grain size 1–2 mm, obtained by sieving the soil through a system of sieves with mesh sizes of 2 and 1 mm, was used for assessing water stability of soil aggregates, easily extractable glomalins and total glomalins.

### Soil analyses

The wet–sieving method of Kandeler^34^ was used to assess the water stability of 1–2 mm soil aggregates. It was performed using HERZOG laboratory equipment (Adolf Herzog GmbH, Vienna, Austria) with sieving time of 5 min and 3 repetitions per sample.

Soil pH was assessed in a water slurry (1:5, w:v) following shaking of the samples for 1 h.

Short- and long-term changes in soil organic matter properties were assessed using analysis of mid-infrared peaks obtained from Fourier transform infrared (FTIR) spectroscopy. For the FTIR analysis, the soil samples (300 mg) were mixed with 900 mg potassium bromide (FTIR grade 99%, Aldrich, Germany) and then ground in an agate mortar. The homogenous mixture was transferred to a diffuse reflectance cup (12 mm diameter) without any pressure, then levelled with a glass microscope slide. FTIR spectra were measured using a Thermo Nicolet Avatar 320 FTIR spectrometer (Nicolet, Madison, WI, USA) in a homogeneous mixture of fine soil and FTIR grade potassium bromide (Sigma-Aldrich, Darmstadt, Germany) and then analyzed at absorption bands indicating aliphatic CH_2_ and CH_3_ (3000–2800 cm^−1^) and aromatic COO– and C=C (1660–1580 cm^−1^) functional groups^35^. Following Demyan et al.^35^, we considered the ratio of peak areas associated with these bands as a measure of soil organic C decomposition (hereafter termed SOC lability).

Content of HWC was determined according to Körschens et al.^36^. Total organic C, N, and S were evaluated using a Vario/CNS analyzer (Elementar Analyser GmbH, Langenselbold, Germany).

Glomalins were extracted from the soil according to Wright and Upadhyaya^37^ by autoclaving in neutral or alkaline citrate solution to yield easily extractable or total fractions and then quantified using the nonspecific colorimetric assay according to Bradford^38^.

### Molecular analyses

To quantify the abundance of total AMF, eukaryotes (including protists), and eubacteria, we employed qPCR with standard primers. First, we extracted DNA from dried soil samples (0.25 g each) using the Power Soil DNA isolation kit (MO BIO Laboratories, Carlsbad, CA, USA) according to the manufacturer’s instructions. We employed the internal DNA standard to check for the presence of PCR inhibitors and to estimate DNA losses during the extraction. To this end, the linearized plasmid carrying fragment of cassava mosaic virus DNA was used^39^. Second, we used the Luna Universal Probe qPCR Master Mix (New England Biolabs, Ipswich, MA, USA) and white FrameStar plates (Institute of Applied Biotechnologies, Prague, Czech Republic). We employed the following primer pairs: NS31: TTG GAG GGC AAG TCT GGT GCC^40^, AML2: GAA CCC AAA CAC TTT GGT TTC C^41^ (AMF); V4_F: CCA GCA SCY GCG GTA ATT CC^42^, V4_R: ACT TTC GTT CTT GAT YRA^42^ (Eukaryota, including protists); Eub338: ACT CCT ACG GGA GGC AGC AG^43^, Eub518: ATT ACC GCG GCT GCT GG^44^ (eubacteria). Primers were synthesized and HPLC purified in Generi Biotech (Hradec Králové, Czech Republic). Internal standard reactions were included. The reaction conditions are described in Table 3.

**Table 3:** Amplification conditions for the different groups of targeted organisms.

Targeted group of organisms	Amplification conditions
AM fungi (Glomeromycotina)	95 °C-3 min, 55 cycles, 95 °C-10 s, 60 °C-15 s, 72 °C-25 s
Eubacteria	95 °C-3 min, 55 cycles, 95 °C-10 s, 55 °C-20 s, 72 °C-25 s
Eukaryota, including protists	95 °C-3 min, 55 cycles, 95 °C-10 s, 48 °C-40 s, 72 °C-60 s
Internal DNA standard	95 °C-5 min, 50 cycles, 95 °C-10 s, 50 °C-20 s, 72 °C-10 s

### Statistical analyses

We first addressed the question whether there is significant variation in stability of soil aggregation (arcsine-transformed) among differently fertilized soils using analysis of variance (ANOVA) and with *p* < 0.05 as the significance cutoff level. This was calculated in the R 3.6.2 statistical environment (R Core Team, 2013, http://www.R-project.org/) after checking for data conformity with ANOVA assumptions (i.e., normality and homogeneity of variances). Two-way ANOVA with factors locality and type of fertilizer was performed. Post-hoc comparisons were carried out using Tukey’s honestly significant difference tests. Mean values and standard errors per treatment combination are presented.

Second, we tested how well would the variation in the assessed soil parameters be explained by the stability of soil aggregates (arcsine-transformed). This was achieved using multivariate statistical methods (redundancy analysis, RDA) supported by Monte Carlo permutation tests with significance estimates adjusted using the false discovery rate approach^45^ carried out in Canoco 5 software^46^. Finally, we used RDA to test if the variations in the soil properties could be explained by the differences among the variously fertilized soils and further possibly to explain soil aggregation. Abundances of soil organisms, HWC, parameters describing organic matter, and S concentration were log-transformed. Concentrations of glomalins, N, and C were square-root transformed prior to the analyses.

## Supplementary Information

Supplementary Information.
