# Supplementary material for: Organic fertilization improves soil aggregation through increases in abundance of eubacteria and products of arbuscular mycorrhizal fungi
Source: Sci Rep. 2021 Jun 15;11:12548. doi: 10.1038/s41598-021-91653-x (PMC8206353; doi:10.1038/s41598-021-91653-x)
Supplement: Supplementary file 1 — Supplementary Information. [file 41598_2021_91653_MOESM1_ESM.pdf]

Supplementary Material

Organic fertilization improves soil aggregation through increases in abundance of eubacteria and products of arbuscular mycorrhizal fungi

Veronika Řezáčová[*,1], Alena Czakó[1], Martin Stehlík[1], Markéta Mayerová[1], Tomáš Šimon[1], Michaela Smatanová[2], Mikuláš Madaras[1]

[1]Crop Research Institute, Drnovská 507, Prague 6, Czech Republic

[2]Central Institute for Testing and Supervising in Agriculture, Hroznová 63, Brno, Czech Republic

*Author for correspondence:

Veronika Řezáčová; Tel: +420 233 022 273; email: rezacova@vurv.cz; ORCID: 0000-0002-1749-0355

Table S1 The composition of the used organic fertilizers. Doses of PK fertilizers approximated local recommendations.

	Dry biomass (%)	C:N	Total N (%)	Combustible substances (%)	P (%)	K (%)
Compost	32.2	11.4	1.30	29.7	1.37	1.64
Digestate I	6.1	6.2	6.38	77.8	1.25	4.52
Digestate II	5.4	3.8	9.67	71.5	1.11	8.00

Fig. S1 Experimental design.

Numbers indicate various treatments, among them 1) mixture of ammonium nitrate and finely ground limestone (LAV, Lovochemie as, 27% N), 2) digestate I (corn silage, cattle slurry), 3) digestate II (corn silage, pig slurry , barnyard manure, hay), 4) compost, and 5) unfertilized control. Ja, Li and Sv are the designations of three different localities. For operational reasons, typical randomization was not used. For easy application of organic fertilizers, the plots are placed one behind the other in a belt. Protective lanes surround each of the plots to eliminate the influence of neighboring plots.

Sv	1	2	3	4	5
	2	1	3	4	5
	1	2	3	4	5
	2	1	3	4	5
Li	1	2	3	4	5
	2	1	3	4	5
	1	2	3	4	5
	2	1	3	4	5
Ja	1	2	3	4	5
	2	1	3	4	5
	1	2	3	4	5
	2	1	3	4	5

Fig. S2 Ordination diagram showing results of redundancy analysis testing the prediction capacity of the type of fertilizer on the assessed soil parameters. The angle between predictor arrow and particular soil parameter arrow approximates the linear correlation between the two variables (proportional to the cosine of the angle). The first (horizontal) axis explains 20.2 % of the total variation in soil parameters, the second axis explains 20.9 % of the total variation.
Arbuscular mycorrhizal fungi (AMF); functional groups (FG); hot water extractable carbon (HWC); easily extractable glomalins (EE glomalin); SOC (soil organic carbon) lability

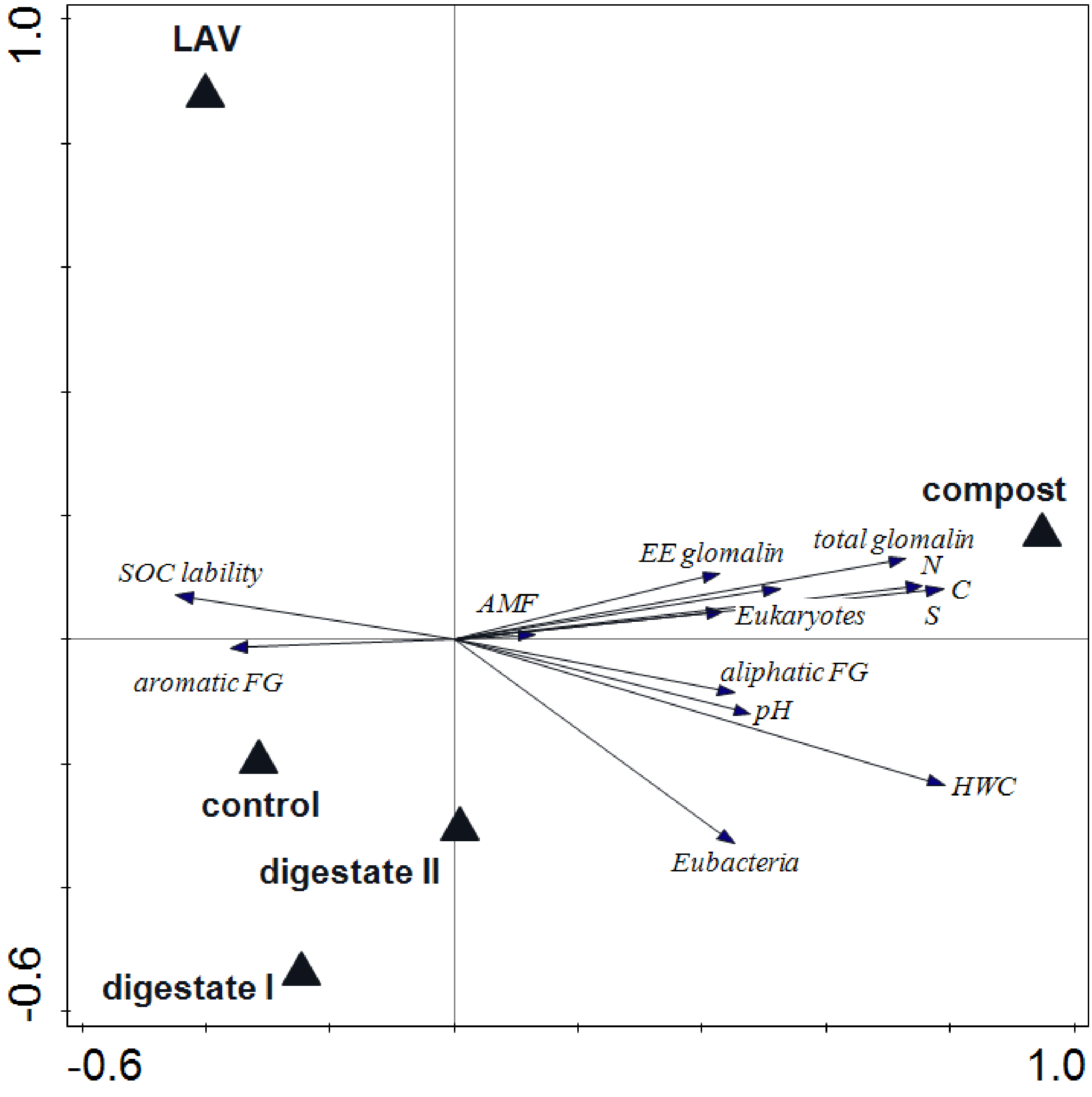

Fig. S3 T-value biplot for the relationships between the soil parameters and the digestate II application. The arrows fully falling within a Van Dobben circle (solid line) indicate a significant positive ($P < 0.05$) relationship between a soil parameter and the predictor. Not all soil parameters located outside the circles are displayed, although they were included in the analysis.
Hot water extractable carbon (HWC)

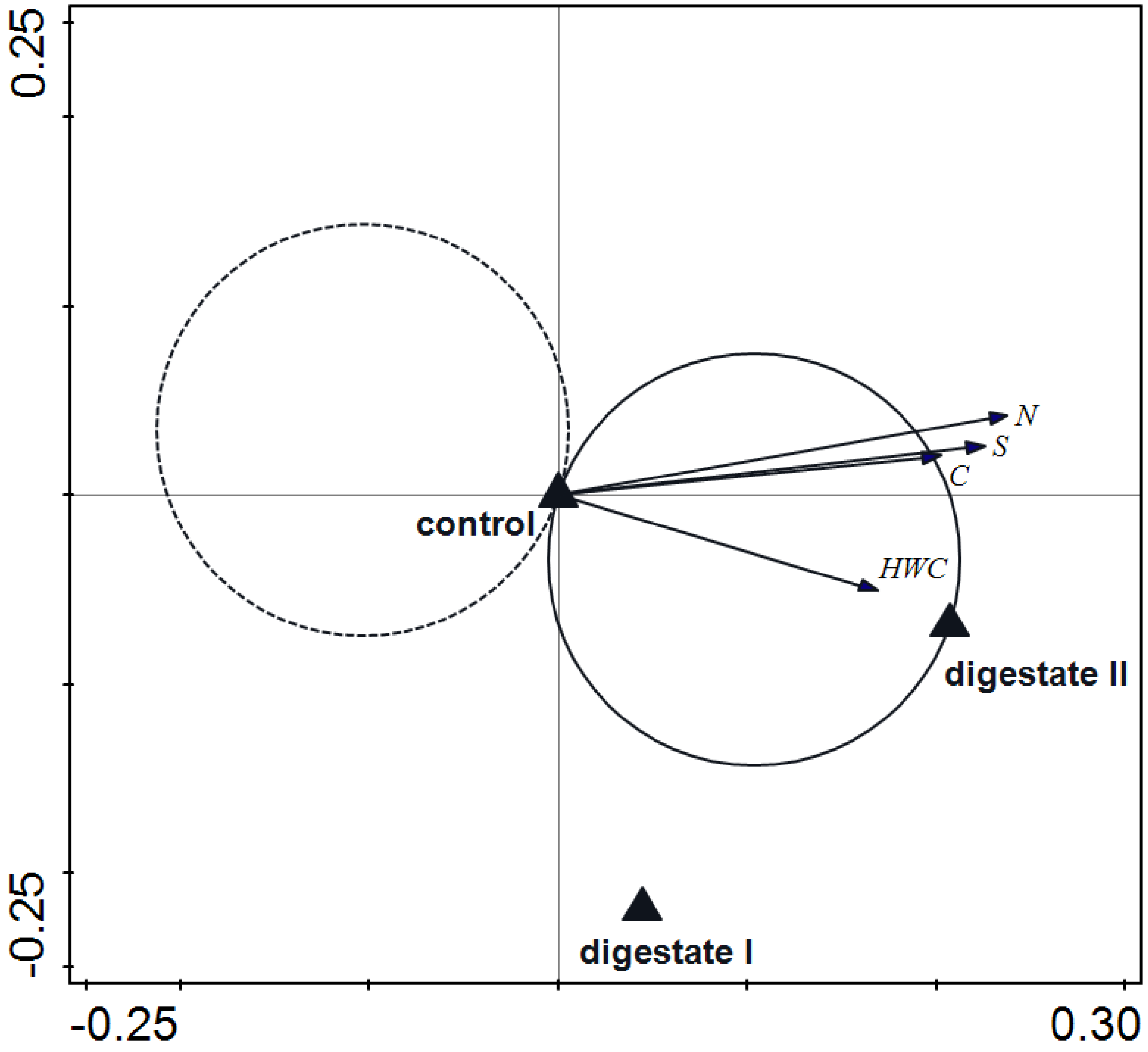

Fig. S4 T-value biplot for the relationships between the soil parameters and the stability of soil aggregates. The arrows fully falling within a Van Dobben circle indicate a significant positive ($P < 0.05$) relationship between the values of a soil parameter and the respective predictor. Not all soil parameters located outside the circles are displayed, although they were included in the analysis. functional groups (FG); hot water extractable carbon (HWC); easily extractable glomalins (EE glomalin); SOC (soil organic carbon) lability

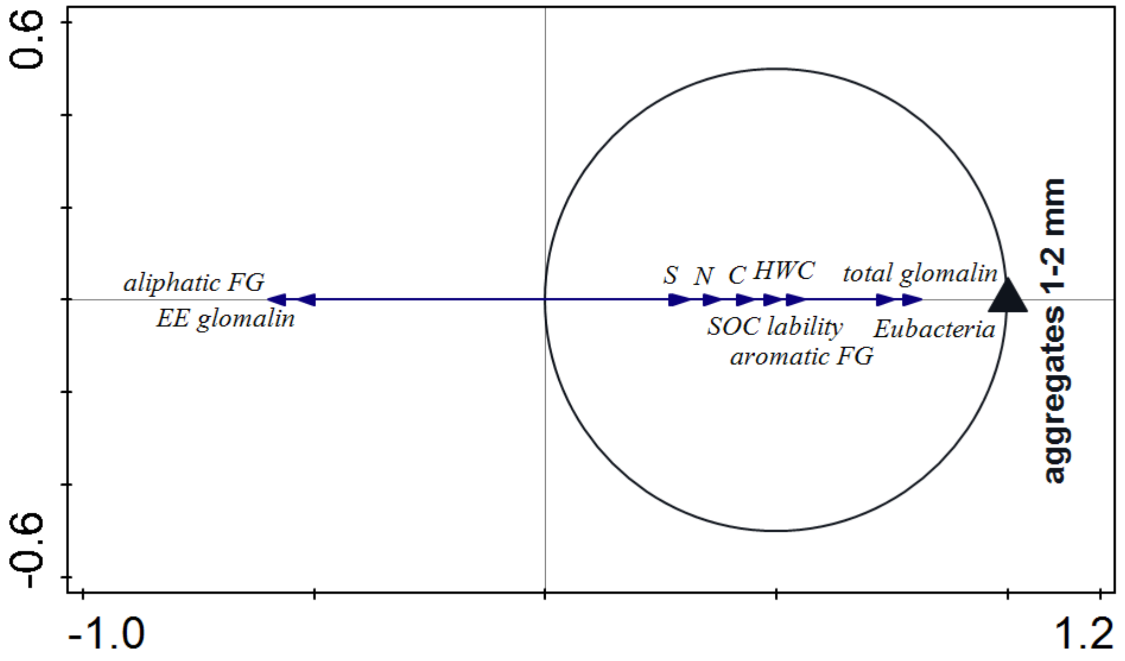

Table S2. Raw data table. Characteristics of the examined field soil samples: percentages of water stable of 1–2 mm soil aggregates; count numbers of eukaryotes including protists, arbuscular mycorrhizal fungi and eubacteria as assessed by real-time PCR; abundance of total glomalins; easily extractable glomalins; concentrations of total organic C, N, and S, and hot water extractable C; aliphatic and aromatic functional groups of soil organic matter, and soil organic C lability assessed using analysis of mid-infrared peaks obtained from Fourier transform infrared (FTIR) spectroscopy and pH measured in aqueous soil suspension.

Article title: Organic fertilization improves soil aggregation through increases in abundance of eubacteria and products of arbuscular mycorrhizal fungi

Authors: Veronika Řezáčová*, Alena Czakó, Martin Stehlík, Markéta Mayerová, Tomáš Šimon, Michaela Smatanová, Mikuláš Madaras

Sample no.	Locality	Type of fertilizer	Stability of soil aggregates 1-2 mm (%)	Eukaryotes incl. protists (CN/1 g)	Arbuscular mycorrhizal fungi (CN/1 g)	Eubacteria (CN/1g)	Total glomalins (mg/g)	Easily extractable glomalins (mg/g)	pH	N (%)	C (%)	S (%)	Hot water extractable C (mg C/g)	Aliphatic functional groups of soil organic matter	Aromatic functional groups of soil organic matter	Soil organic C lability
1	Jaroměřice nad Rokytkou	control	29.08892	7923540536	393556711	59146880786	1.8545	0.527	7.1	0.124	1.27	0.021	0.304688	0.765333	3.869666667	5.094552
2	Jaroměřice nad Rokytkou	control	31.82768	6893163638	350667253	65788335796	1.8265	0.466333	6.74	0.127	1.305	0.021	0.332262	0.690667	3.789333333	5.55437
3	Jaroměřice nad Rokytkou	control	29.74424	10829631594	1.502E+09	65055174941	1.8765	0.518	6.65	0.128	1.261	0.021	0.327437	1.043667	3.794666667	3.633174
4	Jaroměřice nad Rokytkou	control	32.45993	6299383952	3.741E+09	59685061030	1.0675	0.389667	6.64	0.12	1.205	0.02	0.306756	0.425333	4.029333333	10.19939
5	Jaroměřice nad Rokytkou	LAV	31.87349	14885881945	4.131E+09	45046620071	1.8135	0.486667	6.3	0.14	1.356	0.022	0.283319	0.859	4.217	4.909328
6	Jaroměřice nad Rokytkou	LAV	29.79355	10683397953	1.33E+09	59995451743	2.0885	0.48	6.3	0.137	1.336	0.022	0.295038	0.613667	3.803	6.205785
7	Jaroměřice nad Rokytkou	LAV	32.32132	5904773215	2.672E+09	65074460153	1.851	0.408667	6.4	0.14	1.273	0.022	0.304688	0.506333	3.982	8.363446
8	Jaroměřice nad Rokytkou	LAV	33.04233	4626869675	705760947	61441792000	1.843	0.438	6.3	0.135	1.248	0.022	0.278493	0.586333	4.165333333	7.117395
9	Jaroměřice nad Rokytkou	digestate I	39.51895	9199543316	2.19E+09	68814739236	1.914	0.442	5.65	0.129	1.281	0.022	0.360525	0.657	4.143666667	6.313574
10	Jaroměřice nad Rokytkou	digestate I	48.13951	6839833870	1.893E+09	70848688503	1.5835	0.407333	6.64	0.134	1.327	0.023	0.392234	0.616333	4.107666667	6.680984
11	Jaroměřice nad Rokytkou	digestate I	55.21801	8863626065	233590779	86342664921	1.5045	0.419667	6.9	0.129	1.29	0.023	0.331573	0.625333	4.243	6.821615
12	Jaroměřice nad Rokytkou	digestate I	47.2826	8364467241	1.199E+09	83364407949	1.2025	0.404667	6.77	0.128	1.264	0.023	0.33433	0.765333	4.015	5.284245
13	Jaroměřice nad Rokytkou	digestate II	44.4493	6717300371	5.854E+09	62746070534	2.174	0.430333	6.8	0.138	1.346	0.023	0.390856	0.570333	3.811333333	6.778698
14	Jaroměřice nad Rokytkou	digestate II	46.45111	4940497049	3.558E+09	72948160232	1.551	0.419667	6.9	0.146	1.398	0.025	0.360525	0.363667	3.721	10.58146
15	Jaroměřice nad Rokytkou	digestate II	49.58203	10203746362	3.797E+09	63495539229	2.002	0.385	6.95	0.141	1.322	0.024	0.381205	1.108333	3.812666667	3.463348
16	Jaroměřice nad Rokytkou	digestate II	56.65179	16635843979	1.01E+09	54278074186	1.32	0.375333	6.8	0.13	1.233	0.023	0.363972	0.353333	3.936333333	12.27666
17	Jaroměřice nad Rokytkou	compost	41.77645	7044639291	760504613	71758718186	1.879	0.470667	7.2	0.205	2.181	0.029	0.530792	1.435667	3.519	2.463122
18	Jaroměřice nad Rokytkou	compost	46.60596	23590284258	2.241E+09	76040793668	3.0475	0.482333	7.19	0.202	2.176	0.044	0.53217	1.234333	3.549333333	2.894134
19	Jaroměřice nad Rokytkou	compost	51.48735	10521059796	3.162E+09	66982966456	1.7295	0.495667	7.4	0.282	3.131	0.037	0.497703	1.051667	3.603333333	3.483419
20	Jaroměřice nad Rokytkou	compost	50.75694	7387929674	370636344	75198949021	2.42	0.501667	7.43	0.227	2.423	0.032	0.541821	1.397	3.646	2.609885
21	Lípa	control	23.46958	6372208057	294502584	86428316127	1.0345	0.647667	6.4	0.104	1.149	0.018	0.300351	1.049	3.393666667	3.253609
22	Lípa	control	15.28709	4749055439	954271839	64975108804	0.905	0.660333	5.98	0.097	0.992	0.016	0.297547	1.010333	3.452	3.479568
23	Lípa	control	17.03063	10663433335	1.753E+09	73371006816	0.7225	0.625667	5.97	0.103	1.053	0.017	0.302453	0.922333	3.635666667	4.057435
24	Lípa	control	18.16029	10927885699	204290455	61054936368	1.4435	0.638667	6.03	0.105	1.117	0.018	0.263902	1.000667	3.165333333	3.285748
25	Lípa	LAV	21.2744	7648440289	2.587E+09	69213626299	1.4445	0.676667	6.6	0.105	1.122	0.02	0.284229	1.087	3.166	2.935669
26	Lípa	LAV	19.39009	9738279344	511599646	48302646643	0.8715	0.609667	6.2	0.103	1.06	0.018	0.273715	0.979667	3.206	3.287737
27	Lípa	LAV	15.76016	10981091545	975093514	39830998068	1.807	0.626333	6.1	0.098	1.047	0.017	0.234463	0.992667	3.547333333	3.575411
28	Lípa	LAV	27.07723	9921118265	2.526E+09	34179332724	1.4065	0.554667	5.8	0.106	1.082	0.019	0.276519	1.454333	3.424333333	2.356246
29	Lípa	digestate I	23.7007	12254614296	1.28E+09	89992357951	1.647	0.626333	6.45	0.119	1.268	0.019	0.426519	1.427333	3.295333333	2.308366
30	Lípa	digestate I	22.63434	5663695777	1.811E+09	96587395861	1.7455	0.518333	6.45	0.13	1.373	0.021	0.350818	1.214	3.639666667	3.030584
31	Lípa	digestate I	23.22009	6566451915	1.059E+09	84918337133	0.853	0.546667	6.4	0.113	1.202	0.019	0.343809	1.22	3.519333333	2.901876
32	Lípa	digestate I	20.61963	6280060055	323047438	81615700788	1.5335	0.525	6.1	0.115	1.221	0.019	0.346612	1.307667	3.160666667	2.431339
33	Lípa	digestate II	37.37651	16301319302	1.221E+09	1.01468E+11	2.3315	0.609	6.25	0.13	1.368	0.021	0.39007	1.586667	3.014	1.901614
34	Lípa	digestate II	30.27265	16575147638	4.587E+09	91237742537	1.9485	0.572667	6.45	0.125	1.331	0.02	0.379556	1.358667	3.414333333	2.52213
35	Lípa	digestate II	31.70927	13441943675	7.862E+09	69708973568	1.1725	0.654333	6.35	0.125	1.295	0.02	0.366939	1.311667	3.509	2.690831
36	Lípa	digestate II	28.93662	16107669794	4.039E+09	1.0623E+11	1.7195	0.623	6.5	0.121	1.281	0.02	0.34521	1.025667	3.559333333	3.482828
37	Lípa	compost	27.87536	8954110882	5.501E+09	91152466001	1.996	0.745333	6.66	0.176	2.045	0.027	0.67465	2.903333	3.065666667	1.058173
38	Lípa	compost	28.30374	13794921860	2.676E+09	1.16231E+11	1.7345	0.945333	7.12	0.172	1.94	0.027	0.527454	2.649	3.307	1.252233
39	Lípa	compost	31.89801	15466236277	8.984E+09	1.07698E+11	1.9485	1.093333	7.23	0.165	1.836	0.026	0.459463	2.547667	3.413333333	1.341679
40	Lípa	compost	27.83962	7313800503	571986264	89099119750	1.3905	0.984	7.17	0.164	1.851	0.026	0.462267	1.351333	3.705	2.747646
41	Hradec nad Svitavou	control	20.86374	10773167407	2.228E+09	46617931119	1.847	0.522333	6.5	0.101	1.028	0.025	0.322868	2.043333	3.149333333	1.54326
42	Hradec nad Svitavou	control	18.76763	3788895468	1.466E+10	60863500196	1.7375	0.523333	7.28	0.104	1.079	0.016	0.322868	1.755667	3.334333333	1.904618
43	Hradec nad Svitavou	control	16.55075	4922805262	2.661E+09	54310259767	1.2595	0.591667	7.38	0.101	1.012	0.016	0.290894	1.750333	3.095	1.767603
44	Hradec nad Svitavou	control	14.22885	6580232265	2.628E+09	51794939688	1.6325	0.521333	7.35	0.101	0.995	0.016	0.276297	1.579333	3.663	2.335548
45	Hradec nad Svitavou	LAV	19.96127	4167465442	1.635E+09	35507127645	0.9385	0.612667	7.12	0.108	1.038	0.017	0.318698	0.945667	3.342	3.556078
46	Hradec nad Svitavou	LAV	21.01565	5256468657	175024257	41847776072	1.34	0.605333	7.4	0.119	1.105	0.019	0.320088	0.990667	3.373666667	3.459583
47	Hradec nad Svitavou	LAV	17.70904	3627969928	282204893	45908810800	1.78	0.509333	7	0.119	1.068	0.018	0.296455	1.606667	3.687666667	2.305347
48	Hradec nad Svitavou	LAV	17.17309	4168708365	2.693E+09	40859651289	1.6405	0.495	6.55	0.106	1.003	0.017	0.276993	0.976333	3.466666667	3.612433
49	Hradec nad Svitavou	digestate I	25.74209	6489912467	1.175E+09	48698832431	1.5455	0.648667	7.3	0.102	1.005	0.017	0.359708	1.901667	3.410333333	1.794825
50	Hradec nad Svitavou	digestate I	25.83346	6056654248	2.25E+09	36548482069	1.499	0.670667	7.5	0.107	1.077	0.018	0.412535	1.850333	3.423666667	1.857154
51	Hradec nad Svitavou	digestate I	19.19277	5969069444	2.288E+09	36583706185	1.7345	0.645	7.55	0.099	1.005	0.017	0.314527	1.798333	3.374666667	1.880556
52	Hradec nad Svitavou	digestate I	16.56891	2212486383	275181087	55619544266	1.5535	0.645	7.18	0.105	1.052	0.018	0.318698	2.009333	3.319666667	1.653443
53	Hradec nad Svitavou	digestate II	30.06667	7517056536	496084650	51010849994	1.4635	0.537333	7.5	0.101	1.018	0.017	0.318698	2.133667	3.269333333	1.534151
54	Hradec nad Svitavou	digestate II	33.10973	12401997437	189970458	53377230242	1.5515	0.570667	7.4	0.104	1.069	0.019	0.366311	2.335667	3.182666667	1.362916
55	Hradec nad Svitavou	digestate II	27.66766	16291600093	297100890	32923722780	1.766	0.462333	7.3	0.107	1.076	0.019	0.361446	1.806	3.228666667	1.791791
56	Hradec nad Svitavou	digestate II	24.34024	16160665647	731177887	76806644633	1.658	0.472667	7.55	0.097	0.974	0.017	0.324606	1.883333	3.199666667	1.699284
57	Hradec nad Svitavou	compost	30.09405	9241536348	1.204E+09	73526769815	2.0555	0.714667	7.11	0.128	1.364	0.036	0.369787	2.110667	2.896	1.375556
58	Hradec nad Svitavou	compost	29.32517	17583900574	1.109E+09	69708973568	2.4205	0.714333	7.41	0.137	1.437	0.034	0.430955	2.438	2.689666667	1.102521
59	Hradec nad Svitavou	compost	27.72282	2.82036E+14	2.537E+09	72658897631	2.5015	0.72	7.57	0.14	1.48	0.022	0.319045	2.256	2.860333333	1.269478
60	Hradec nad Svitavou	compost	25.79738	2.83663E+14	881409013	70567750974	2.678	0.634333	7.59	0.138	1.453	0.022	0.385774	2.079	3.029666667	1.470575
